# The effect of diameter size of single-walled carbon nanotubes on their high-temperature energy storage behaviour in ionic liquid-based electric double-layer capacitors†

**DOI:** 10.1039/d0ra08579k

**Published:** 2020-11-11

**Authors:** Ayar Al-zubaidi, Nanami Asai, Yosuke Ishii, Shinji Kawasaki

**Affiliations:** Department of Life Science and Applied Chemistry, Nagoya Institute of Technology Gokiso-cho, Showa-ku Nagoya 466-8555 Japan a.al-zubaidi.052@nitech.jp ishii.yosuke@nitech.ac.jp kawasaki.shinji@nitech.ac.jp

## Abstract

We investigated the effect of the diameter size of single-walled carbon nanotubes (SWCNTs), on their high-temperature energy storage behavior in an electric double layer capacitor (EDLC) using the ionic liquid triethyl(2-methoxyethyl) phosphonium bis(trifluoromethylsulfonyl)imide (P_222(2O1)_-TFSI). We used four SWCNT samples with diameter sizes ranging from 0.8 to 5 nm, and evaluated their electrochemical charge storage behavior through galvanostatic charge/discharge and electrochemical impedance spectroscopy (EIS). We found that for the SWCNTs with small average diameter of 1 nm, the value of the electrode capacitance measured at a current density of 5 mA g^−1^ increased from 15.8 at room temperature to 27.5 F g^−1^ at 150 °C, and the value measured at a current density of 80 mA g^−1^ increased from 14.0 at room temperature to 22.1 F g^−1^ at 150 °C. The larger diameter samples on the other hand did not show any significant change in their capacitance with temperature. We calculated the size of the interstitial tube spaces from the Raman spectra of the samples, and used density functional theory (DFT) calculations to estimate the sizes of the cation and anion of the electrolyte. The obtained results suggest that the temperature-induced changes in the electrolyte properties improved the ion accessibility into the otherwise constrained space inside the small diameter SWCNTs, while the spaces inside the larger SWCNTs already provided easily accessible storage sites hence good performance at room temperature, making the increase in temperature of little to no effect on the charge storage performance in such SWCNTs.

## Abstract 2

Temperature-induced changes in electrolyte improved ion accessibility inside small SWCNTs, while spaces inside larger SWCNTs provided accessible storage sites and good performance at RT, rendering temperature of little effect on their charge storage performance.

## Graphical abstract

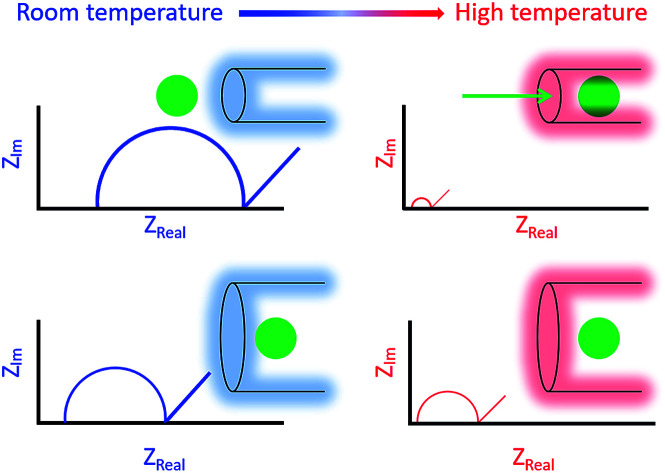

## Introduction

The use of electric double layer capacitors (EDLCs) is being increasingly expanded to many applications, including some which take place under elevated temperatures, like electric and hybrid vehicles, electric aircraft, aerospace electronics, and oil drilling rigs. The use of EDLCs in such applications requires the optimization of their components to operate safely and smoothly under elevated temperatures. Two issues of particular importance in that regard are the reduction in the voltage window of solvent-based solutions, and the safety concerns stemming from the flammability risk in the particular case of organic electrolytes. To address both issues, ionic liquids have long been under investigation as solvent-free alternatives that enjoy the large potential window and the thermal stability needed for safe and efficient energy storage.^1–3^ These attractive properties sparked a large body of research revolving around the use of ionic liquids for electrochemical energy applications, especially those involving elevated temperatures.^4–18^

Electrochemical energy storage devices use electrodes made from porous materials with high surface area provided by a network of pores on the surface of the electrode. These pores offer the space needed for diverse physical and chemical changes to take place, leading to energy storage. In such devices, the energy storage is largely dependent on the structural properties of the electrode material. This raises the logical question of the effect of the structural properties of the electrode on the energy storage performance of the EDLCs at elevated temperatures, and the design factors to be considered in such systems, especially when the device is expected to operate under both elevated temperatures and high charging rates. To the best of the authors' knowledge, this factor has remained largely unaddressed, apart from a study that compared the behaviour of carbon nanotubes to that of nano-onions used as electrodes for EDLCs operated at elevated temperatures.^6^

To address this question in a systematic manner, we used in the present study a number of single-walled carbon nanotube (SWCNT) samples with diameters ranging from 0.8 to 5 nm, and paired them with an ionic liquid to evaluate their behaviour as EDLC electrodes under temperatures up to 150 °C, and explore the influence of the tube diameter on the charge storage under elevated temperatures. The regular and well-defined nature of the porosity of SWCNTs, and the possibility to assess the occurrence of ions storage in specific storage sites in an SWCNTs sample, provide systematic assessment tools to map the electrode behaviour in relation to its diameter size.

## Experimental

We used four samples of SWCNTs, which were denoted SWCNT1.0, SWCNT1.5, SWCNT2.0 and SWCNT5.0, in a general reference to the expected tube size of each sample, which is given in more detail in Table S1 in the ESI† along with other relevant information. For further insight and comparison, we also used a sample of activated carbon with a high specific surface area of 1293 m^2^ g^−1^.

The samples were imaged using scanning electron microscopy (SEM) as shown in Fig. S1,† and their specific surface area and other structural properties were determined from nitrogen adsorption isotherms and Raman spectroscopy (Fig. S2†). Where possible, the tube diameter range for the SWCNTs samples was determined from the frequency of the radial breathing mode (RMB) in the corresponding Raman spectrum of the sample. We chose the ionic liquid triethyl(2-methoxyethyl) phosphonium bis(trifluoromethylsulfonyl)imide (P_222(2O1)_-TFSI) as the electrolyte for considerations given in the ESI section.† The ion sizes for P_222(2O1)_^+^ cation and TFSI^−^ anion were calculated from density functional theory (DFT) calculations performed using Gaussian09 software, using an M06-2X exchange correlation functional and aug-cc-pVDZ basic function. The thermal stability of the electrolyte was evaluated using thermogravimetric analysis (TGA) under nitrogen flow, and its electrochemical stability window at different temperatures was evaluated using linear sweep voltammetry (LSV). The energy storage behaviour of electrolyte using the different samples was investigated through galvanostatic charge discharge (GCD) and electrochemical impedance spectroscopy (EIS). The measurements were conducted in a temperature-controlled chamber at temperatures of 25, 50, 100, 150, 200, 250, and 300 °C. The details of the Experimental procedures are given in the ESI section.†

## Results and discussion

**Fig. 1:**
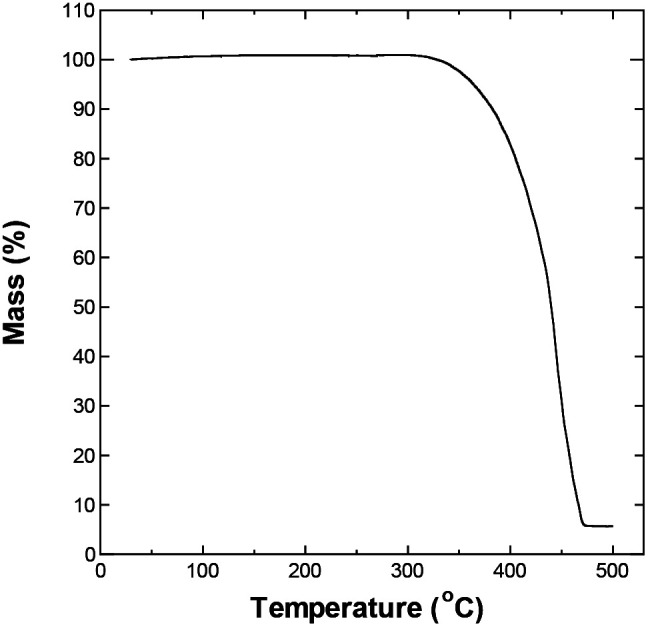
TGA curve for P_222(2O1)_-TFSI measured with heating rate of 5 °C min^−1^ under N_2_ flow.

The upper temperature limit for conducting the experiments was chosen by evaluating the thermal stability of the electrolyte, and its potential window at different temperature. Fig. 1 shows the thermogravimetric analysis (TGA) curve for P_222(2O1)_-TFSI under N_2_ flow. The electrolyte appears to be thermally stable at temperature as high as 300 °C. The temperature corresponding to a mass loss of 10% was around 383 °C, which slightly is lower than the reported limit of 404 °C,^19^ and may be related to the dependence of the onset of decomposition on experimental factors such as the sample weight and heating rate.^20–22^

**Fig. 2:**
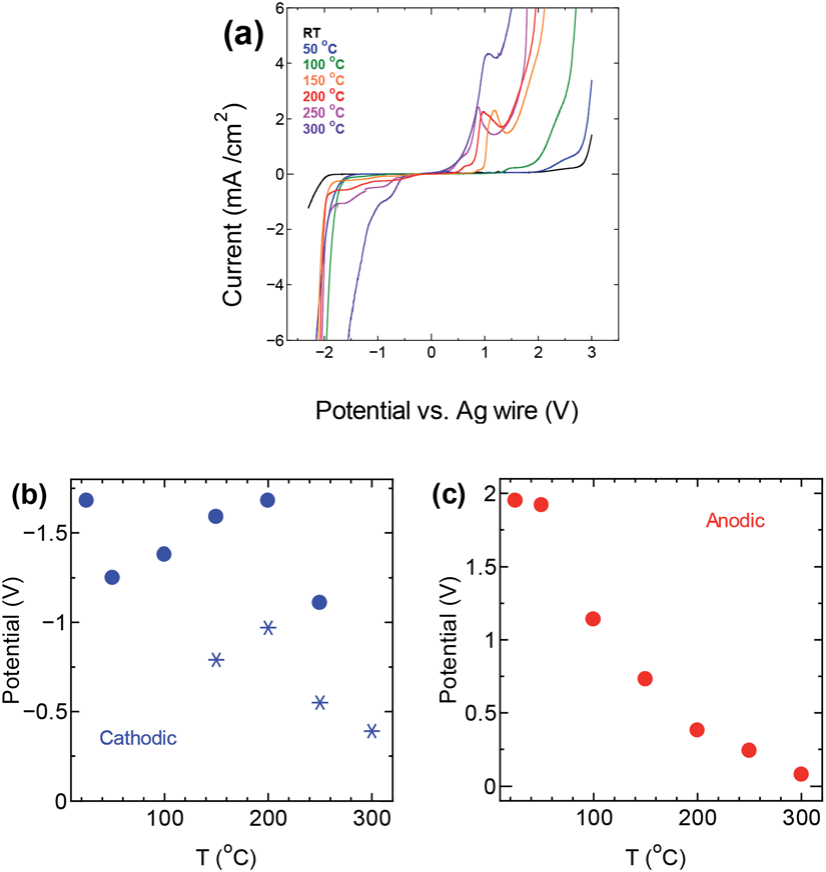
(a) LSV measurements for P_222(2O1)_-TFSI in a three-electrode setting (b) cathodic and (c) anodic limits of the potential stability window of the electrolyte at different temperatures.

The electrochemical potential window of P_222(2O1)_-TFSI was evaluated from LSV measurements at temperatures up to 300 °C, using a three-electrode cell with platinum disk as the working electrode. The results are shown in Fig. 2. The limits of the potential stability window were determined using a conservative variation of the linear fitting method,^23^ by plotting the differential current density-potential (Δ*i*/Δ*E*) against the applied potential, then defining the cathodic/anodic limits as the edges of the respective linear regions observed before the onset of the decomposition reactions (an example is shown in Fig. S3 in the ESI section†). The obtained values are shown in Fig. 2b and c.

The anodic potential limit (Fig. 2c) shows the expected decrease with temperature. The cathodic potential limit on the other hand, followed an upward trend with the stability limit pushed farther away from the open circuit potential up to a temperature of 200 °C, before decreasing again with further increase in temperature, as shown by the solid circles in Fig. 2b. The star points are potential values corresponding to a second peak that appeared at 150 °C and was taken as the potential limit instead of the solid circle points at the same temperature. The upward trend in the potential limits at temperatures below 200 °C is similar to previous observations^24^ that reported the extension of the cathodic limit of the stability potential window of LiTFSI in a lithium ion battery setting, and attributed the extension to the formation of a passivation layer due to the reduction of TFSI-anion. The passivation layer suppresses the hydrogen evolution, pushing its onset farther from the open circuit potential.

The onset of decomposition on the TGA curve suggests that electrolyte remains usable at temperatures up to 300 °C, which is consistent with the obtained values for the stability window, albeit for an impractically narrow potential window of less than 0.5 V. Therefore, we decided to conduct the remaining of our investigation within the conservative upper limit of 150 °C.

**Fig. 3:**
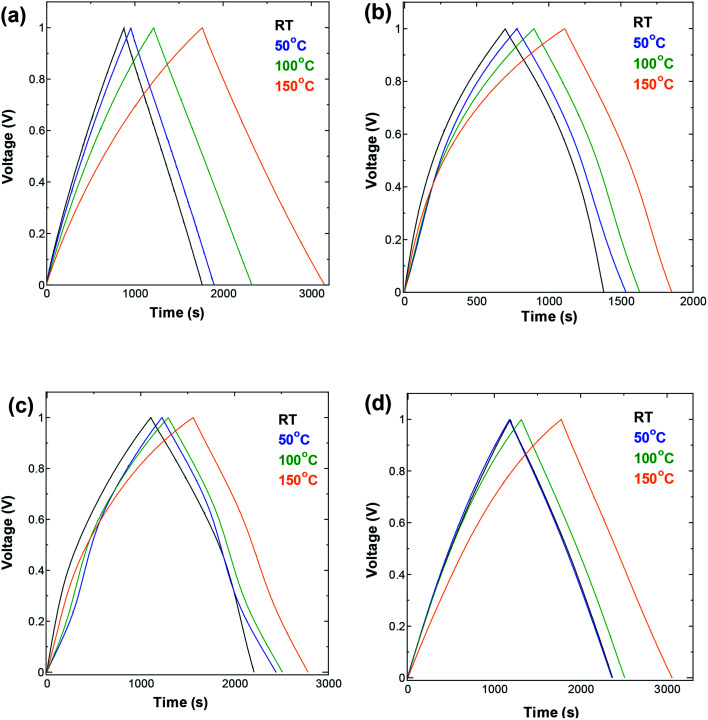
GCD measurements performed at current density 10 mA g^−1^ in a symmetric two-electrode cell using (a) SWCNT1.0 (b) SWCNT1.5 (c) SWCNT2.0, and (d) SWCNT5.0 as electrodes.

We performed galvanostatic charge and discharge GCD measurements (Fig. 3) to a voltage of 1.0 V, using a symmetric two-electrode cell operated at different temperatures and different current densities, with SWCNT1.0, SWCNT1.5, SWCNT2.0, or SWCNT5.0 as the electrode material.

The GCD curves shown in Fig. 3 were measured at current density of 10 mA g^−1^. One can see a visible broadening in the GCD curve measured for SWCNT1.0 (Fig. 3a), and a change in its slope with the increase in temperature, indicating the increase in the obtained capacitance. The other three samples (Fig. 3b–d) did not show pronounced change at temperatures below 150 °C. The AC electrode (Fig. S4a†) showed similar improvement in the capacitance to that of SWCNT1.0. Unlike SWCNTs, AC consists of irregularly shaped pores interconnected in a complex tortuous network which, in combination with its lower crystallinity and electric conductivity, poses additional limitations on the ion storage in AC. The changes induced by increasing the temperature also seemed to eliminate the voltage drop of about 0.15 V that appears on the room-temperature discharge curve (black GCD curve in Fig. S4a†), and gradually diminishes with the increase in temperature. This voltage drop is associated with the internal series resistance that is generally considered to be dominated by electrolyte resistance in both the bulk and in the porosity of the electrode.^8,25,26^ The absence of such voltage drop from the GCD curves of the SWCNTs samples is explained by their superior crystallinity and electric conductivity compared to AC.

**Fig. 4:**
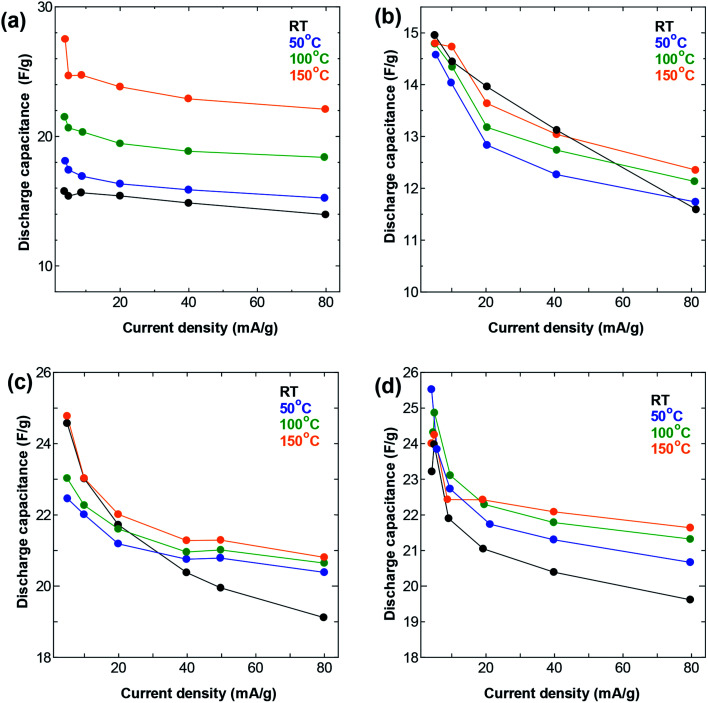
Rate performance at different temperatures in a symmetric two-electrode cell using (a) SWCNT1.0 (b) SWCNT1.5 (b) SWCNT2.0, and (d) SWCNT5.0 as electrodes.

Increasing the temperature also resulted in an improvement in the performance at high current density (Fig. 4), following similar trends as those seen in the GCD measurements. The increase in temperature resulted in visible improvement in the capacitance obtained at all current densities for the SWCNT1.0 sample, but not so much for the other three samples. Again, the AC sample showed similar improvement to that of SWCNT1.0, confirming the beneficial effect of elevated temperature in improving the ion accessibility not only for materials with small pore size, but also for those with disorganized networks of irregularly shaped pores. The capacitance obtained for SWCNT1.0 at current density of 5 mA g^−1^ increased from 15.8 at room temperature to 27.5 F g^−1^ at 150 °C, while the value obtained at 80 mA g^−1^ increased from 14.0 F g^−1^ at room temperature to 22.1 F g^−1^ at 150 °C. The other samples did not show any visible improvement in the high-rate performance, while the capacitance obtained for AC at 80 mA g^−1^ had a drastic increase from 1.9 F g^−1^ at room temperature to 34.4 F g^−1^ at 150 °C (Fig. S4b†). It is interesting to note that the sample SWCNT1.5 had visibly lower capacitance (Fig. 3b and 4b) and smaller changes in the rate performance (Fig. 4b) compared to the other samples within the whole temperature range. We speculate that the usability of the adsorption spaces in SWCNT1.5 is the least optimum among all the samples, which is consistent with previously reported trends that reported the capacitance to decreases with pore size up to certain a critical value, after which it is likely to increase again^27–29^ This critical value will depend strongly on the size of the ions in the electrolyte, and is therefore likely to be a characteristic parameter in a given electrochemical system.

**Fig. 5:**
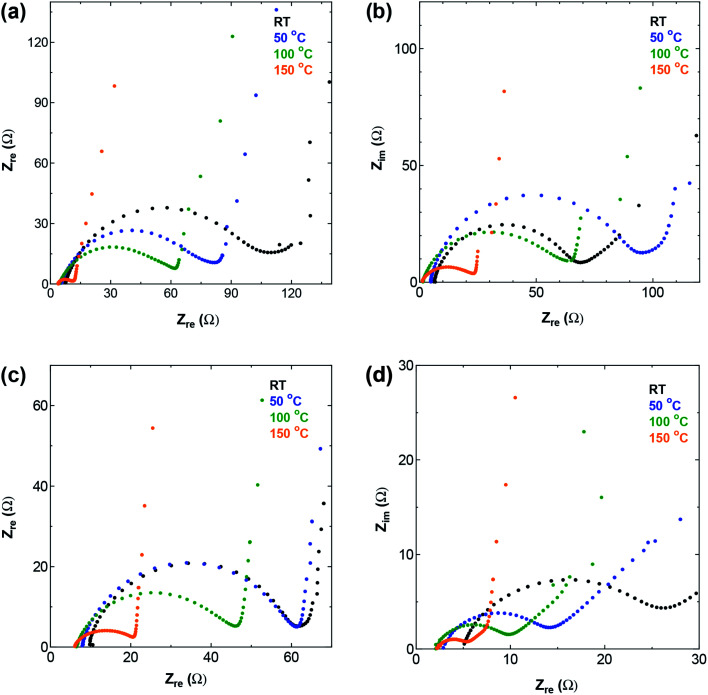
The high and medium frequency region of Nyquist plot for (a) SWCNT1.0 (b) SWCNT1.5 (c) SWCNT2.0, and (d) SWCNT5.0 as electrodes.

Electrochemical impedance spectroscopy (EIS) offers information on the kinetics of charge transfer and double layer formation in porous electrodes at different temperatures, through the changes observed in the three regions of low, medium, and high frequency of the complex impedance graph known as the Nyquist plot. The high and medium frequency regions of the Nyquist plot for the four SWCNT samples and AC are shown in Fig. 5 and S5,† respectively, and the Randles equivalent circuit model used for fitting the data is given in Fig. S6† for SWCNT1.0 at RT as fitting example. The circuit shows the components describing the electrochemical behaviour in the system, and in particular, the significant role of the charge transfer resistance component *R*_C_, which is given by the diameter of the semicircle in the medium-to-high frequency range, and embodies the resistance at the interface between porous surface of the electrode and the electrolyte.^30,31^ The intersection of the impedance spectrum with the real impedance axis (the *X*-axis) on the high frequency side, shows the electric resistance (*R*_e_) stemming from all the connections (measurement connections, electrode-current collector) and the resistance in the bulk electrolyte. In the present work, while the increase in temperature caused *R*_e_ to shift towards less resistive values for all the samples, we did not detect any significant difference between *R*_e_ values for the different SWCNTs samples at any given temperature (results not shown). On the other hand, the value of *R*_C_ showed visible dependence on the sample used. The Nyquist plot for SWCNT1.0 (Fig. 5a) shows the largest charge transfer resistance *R*_C_ at room temperature among the four samples, and the most significant reduction in resistance with temperature. One anomaly we observed was in the EIS curve measured at 50 °C for the sample SWCNT1.5, which shows higher charge transfer resistance (larger semicircle) compared to the curve measured at room temperature, which we could not explain in relation to the inherent properties of the sample, especially considering the absence of similar anomalies on the GCD curve, and the insignificant changes in the rate performance of SWCNT1.5.

The room temperature *R*_C_ value for AC (Fig. S5†) was larger than those of the SWCNT samples, due to the inferior crystallinity and electric conductivity of AC, and the low accessibility of ions into the complex porous network in AC.

The increase in temperature is known to cause a reduction in the viscosity of the electrolyte, which improves the ion mobility and ionic conductivity (as seen in Fig. S7 in the ESI section†), leading to improved charge transfer kinetics.^8,16,32^ However, the larger diameter SWCNT samples showed little to no improvement in the charging discharging behaviour. This suggests that the space available for ion storage in those samples is large enough to ensure good accessibility to ions at room temperatures. A larger space will also allow ions the freedom to reorient themselves upon the increase in potential or current densities. This ion reorientation is particularly relevant in the case with large ions,^33^ and leads to charge redistribution that clears more space for the adsorption of additional ions, and maximizes charge storage in the pores of the electrode.^34,35^ With such smooth accessibility, the pores in the large diameter SWCNT samples should be used more efficiently for ion storage compared to smaller SWCNTs, to the extent that the changes induced by increasing the temperature caused no practical improvement in the energy storage in larger SWCNTs at temperatures below 150 °C.

**Fig. 6:**
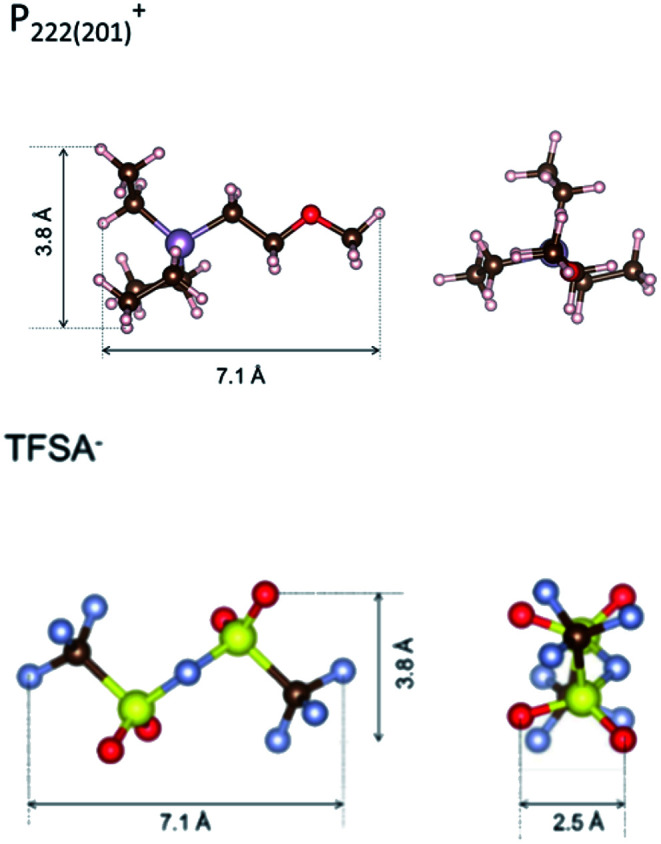
The dimensions of P_222(2O1)_^+^ cation and TFSI^−^ anion, estimated from density functional theory calculations.

**Fig. 7:**
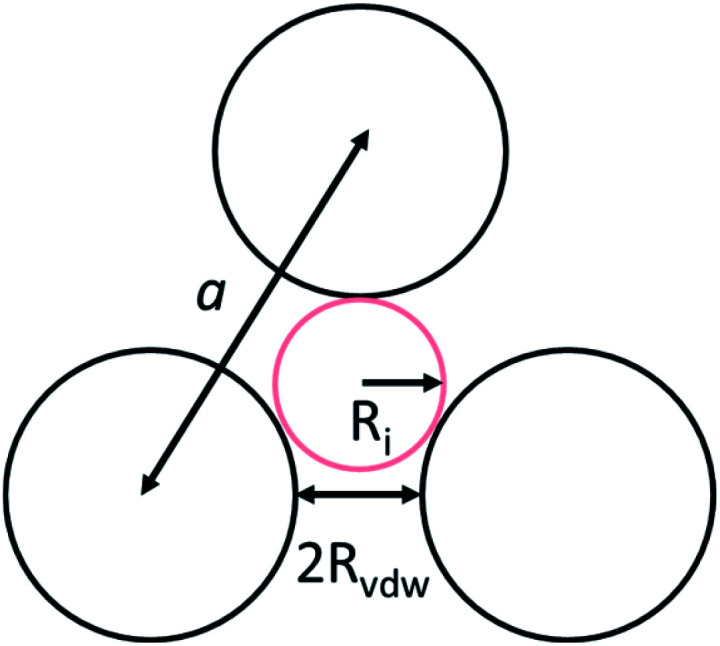
The intertube space in the triangular lattice of a SWCNTs bundle.

To identify the possible sites where the ion storage is likely to have improved, we take a closer look at the porous structure in SWCNTs and the candidate ion storage sites. SWCNTs tend to aggregate under the effect of van der Waals interactions, forming a bundle that may be represented by the pseudo-two-dimensional hexagonal lattice shown in Fig. 7. The bundle structure can be observed by TEM observation, and its corresponding X-ray diffractions can be observed experimentally.^36–38^ The sites available for ion storage in SWCNTs will therefore include the outer surface of the bundled tubes, the interstitial space between the tubes and bundles, and the hollow space inside the tubes. In the case of P_222(2O1)_-TFSI, our DFT calculations resulted in the molecular dimensions shown in Fig. 6. If we assume an approximate van der Waals distance (2*R*_vdw_) of 0.31 nm between the graphitic layers of SWCNTs (which is slightly shorter than the graphite layer–layer distance of 0.335 nm),^36^ the we can calculate the interstitial radius *R*_i_ from the equation:1
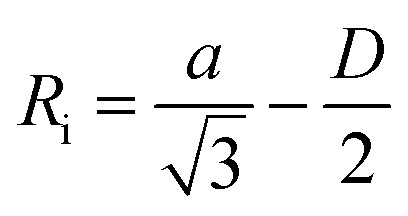


In the equation above, *a* is the hexagonal lattice constant (determined as *a* = 2*R*_vdw_ + *D*), and *D* is the tube diameter (Fig. 7). Using the SWCNT diameters listed in Table S1,† the calculations resulted in interstitial diameter values of 0.45–0.57 nm for SWCNT1.0 and 0.57–0.61 nm for SWCNT1.5, suggesting the difficulty for ions to access into the interstitial space in the two samples, and rendering the space inside the tubes of those samples the main narrow space for which the improvement in ion storage is likely to have been improved by increasing the temperature. The interstitial space should to be larger than 0.79 nm for SWCNT2.0, and around 0.82–1.13 nm for SWCNT5.0, which should allow easier access of the ions of P_222(2O1)_-TFSI into those samples at room temperature. This should explain the visible improvement in the capacitance and high-rate performance, and the drop in *R*_C_ values for SWCNT1.0 with the increase in temperature, and the absence of such visible changes for the larger SWCNT samples.

**Fig. 8:**
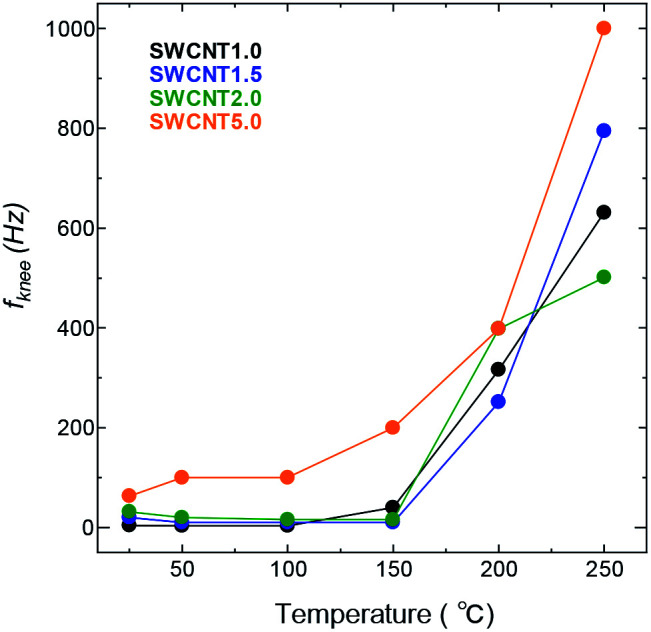
Change in knee frequency with temperature for the SWCNT samples.

Next, we take a look at the transitional part of the low-frequency region of the Nyquist plot, where the increase in frequency causes a transition in the charge storage behaviour from predominantly capacitive (the semi vertical line) towards one where a resistive component becomes relevant to the transfer kinetics, and slows down the ion transport (the 45° line). This transition is marked by the so-called knee frequency (*f*_knee_)^39,40^ identified as the highest possible frequency under which the system can maintain pure capacitive behaviour. Fig. 8 shows that the *f*_knee_ values for all the SWCNT samples increased visibly with temperature, and following roughly the same trend. SWCNT5.0 had the highest *f*_knee_ values in the whole temperature range, due to the larger pore size of SWCNT5.0 posing less constraint to the ion transport as discussed above. The *f*_knee_ values of for AC were an order of magnitude lower than those for other samples, and their change with temperature fluctuated with temperature, so they were disregarded from the comparison.

**Fig. 9:**
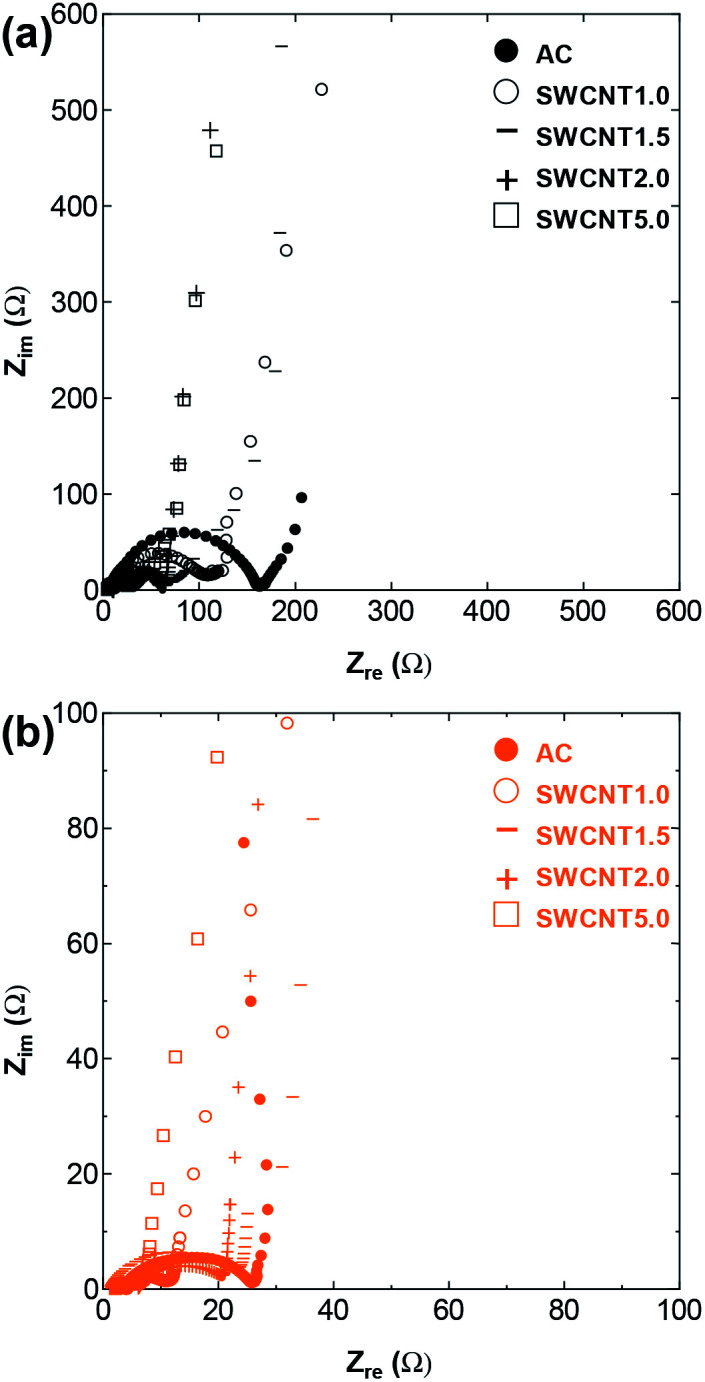
The full-scale Nyquit plot including the low-frequency region for the tested samples at (a) RT and (b) 150 °C.

Fig. 9 expands the Nyquist plot to show the region where the frequency is low enough to detect the role of ion diffusion in the observed impedance. The figure shows the improvement in the capacitive behaviour with temperature for all the samples, evidenced by the increase in the imaginary part and the change in the plot that tends to the vertical line characteristic of capacitive behaviour. Interestingly, the figure shows that the ion diffusion at 150 °C is of the same order for all the samples, which indicates that operating at elevated temperature was effective in improving the performance of samples that suffered from constrained ion storage at room temperature, raising their energy storage efficiency to a level on par with that of samples with inherently efficient ion storage properties.

## Conclusions

We used SWCNTs as electrodes for an ionic liquid-based EDLC operated at elevated temperatures reaching 150 °C, and observed that the increase in temperature improved the energy storage performance and increased the capacitance obtained from SWCNTs with small diameter size (0.8–1.2 nm), while no visible improvement was observed for SWCNTs with larger diameters. The calculated ion and SWCNT sizes suggest that the improvement in energy storage is likely to have been due to the improvement in ion accessibility into the hollow space inside the small diameter of small SWCNTs, which improved the charge storage kinetics to a level comparable to that of larger tubes with inherently better ion accessibility.

## Conflicts of interest

There are no conflicts to declare.

## Supplementary Material

RA-010-D0RA08579K-s001
