# Supplementary material for: The effect of diameter size of single-walled carbon nanotubes on their high-temperature energy storage behaviour in ionic liquid-based electric double-layer capacitors
Source: RSC Adv. 2020 Nov 11;10(67):41209–16. doi: 10.1039/d0ra08579k (PMC9057770; doi:10.1039/d0ra08579k)
Supplement: RA-010-D0RA08579K-s001 [file RA-010-D0RA08579K-s001.pdf]

Electronic Supplementary Material (ESI) for RSC Advances.
This journal is © The Royal Society of Chemistry 2020

# The effect of diameter size of single-walled carbon nanotubes on their high-temperature energy storage behaviour in ionic liquid-based electric double-layer capacitors

Ayar Al-zubaidi,[1]* Nanami Asai,[1] Yosuke Ishii, [1]* Shinji Kawasaki[1]*

## Supporting Information

**Electrode materials**

SWCNTs may contain metallic catalyst particles remaining from the synthesis process. In the present study, SWCNT5.0 which was synthesized by the super growth method did not contain such impurities, while the samples SWCNT1.5 and SWCNT2.0 two samples were purified to remove the metallic impurities by acid treatment using 10% HCl. SWCNT1.0 was excluded from the acid treatment to avoid the damaging of the small-size tubes. Prior to the acid treatment, the samples SWCNT1.5 and SWCNT2.0 underwent heat treatment in air at 350 °C for 30 minutes, to remove amorphous carbon.

The samples SWCNT1.0, SWCNT1.5, and SWCNT2.0 were then annealed to remove any chemical groups and close any defects on their surfaces. The annealing treatment was performed at 1100 °C for 14 hours under vacuum. Then, the samples underwent heat treatment in air to remove the caps of the tubes before using them. The temperature needed for this decapping treatment was determined from thermogravimetric (TGA) analysis of the samples. The analysis was performed using a SHIMADZU TGA-50 analyzer, and the samples were heated to 1000 °C at a rate of 10 °C /min, under dry air flow of 100 mL /min. The temperatures for decapping were determined to be 270 °C for SWCNT1.0, 480 °C for SWCNT1.5, and 550 °C for SWCNT2.0.

The samples were imaged by scanning electron microscopy (SEM) using a JEOL JSM-6010LA microscope operated at 10 kV. The obtained images are shown in Fig. S1.

The specific surface area of the obtained samples was evaluated from nitrogen adsorption isotherms performed using a SHIMADZU Gemini2375 analyzer. The surface area was determined using the Brunauer–Emmett–Teller (BET) method. The surface structure of the samples was further characterized using Raman spectroscopy. The spectra were collected using a JASCO NRS-3300 spectrometer, with 532 nm wavelength Nd:YAG laser as the excitation source. The obtained spectra are shown in Fig. S2.

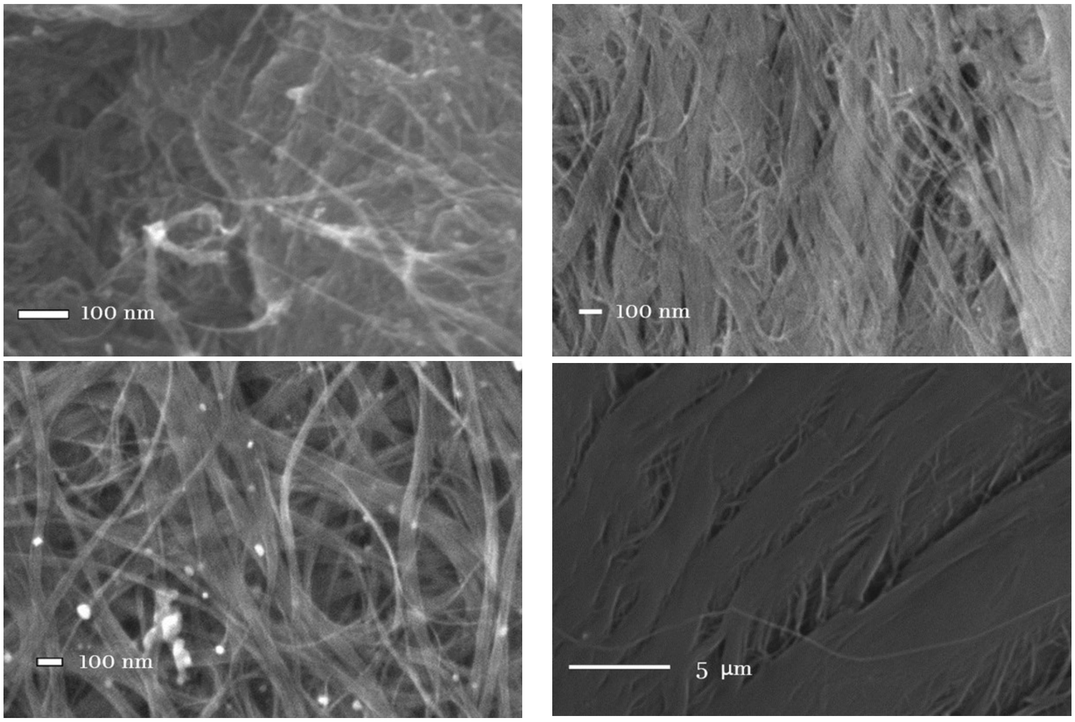


Fig.S1 SEM images for (a) SWCNT1.0 (b) SWCNT1.5 (c) SWCNT2.0 and (d) as-received SWCNT5.0

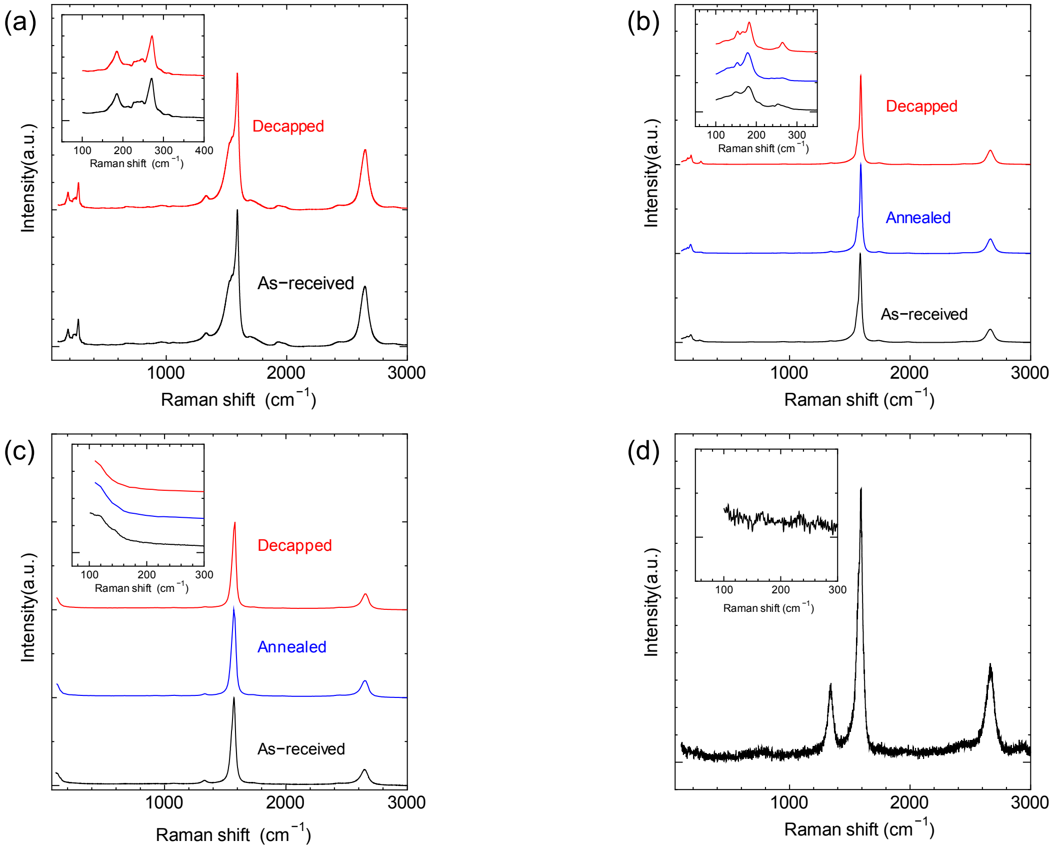


Fig.S2 Raman spectra for (a) SWCNT1.0 (b) SWCNT1.5 (c) SWCNT2.0 and (d) as-received SWCNT5.0

The diameter range for a given SWCNT sample was obtained from the frequency of the radial breathing mode (RBM), using the equation:

$$\omega_{RBM} = \frac{A}{D} + B \qquad \ldots S1$$

In equation S1, $\omega_{RBM}$ is the frequency of the radial breathing mode seen in the low frequency region of the Raman spectra (the insets in Fig.S2), A = 234 $cm^{-1}$, and B is the upshift in $\omega_{RBM}$ assigned to tube-tube interactions, and has the value 10 $cm^{-1}$.[1] In the present study, and due to the limitations imposed by the experimentally measurable range of the spectrometer, the RBM region of the sample SWCNT2.0 showed only a partial peak with no defined maximum in the measurable frequency limits, while that of the SG appears to have been well outside the measurable limits of the device, as seen from the flat spectrum in Fig. S2d. Therefore, we only made a rough estimation of the diameter range for SWCNT2.0, and used the diameter range information supplied by the manufacturer for SWCNT5.0. The calculated diameter values can be used to obtain the candidate chiralities for the SWCNTs in each sample. The structural properties obtained for all the samples are summarized in Table S1.

Table S1. Properties and surface structure of the electrode materials

| Sample (and product name) | Maker | BET surface area ($m^2/g$) | Diameter (nm) | Candidate chiralities (n,m) |
|---|---|---|---|---|
| SWCNT1.0 (HiPco® Purified) | NanoIntegris | 730 | 1.34 | (13,6) |
| | | | 1.07 | (11,4), (13,1) |
| | | | 0.98 | (9,5), (10,4), (12,1) |
| | | | 0.89 | (7,6), (10,2) |
| SWCNT1.5 (EC1.5) | Meijo Nanocarbon Co., Ltd. (eDIPS method) | 970 | 1.63 | (16,7), (17,6), (19,3), (20,1) |
| | | | 1.51 | (11,11), (12,10), (13,9), (16,5), (18,2) |
| | | | 1.36 | (14,5), (16,2) |
| SWCNT2.0 (EC2.0) | Meijo Nanocarbon Co., Ltd. (eDIPS method) | 1090 | > 2.6 | N/A |
| SWCNT5.0 (ZEONANO®SG101) | Zeon Nano Technology (super growth method) | 831 | 3-5 | N/A |
| AC (activated carbon, YP-50F) | Kuraray Co., Ltd. | 1293 | | N/A |

**The Electrolyte**

The evaluation of the performance of an EDLC cell capable at high-temperature operation requires the use of an electrolyte that is stable at high temperatures. Ionic liquids have high thermal stability, but high viscosity and poor conductivity. In this study, triethyl (2-methoxyethyl) phosphonium bis (trifluoromethylsulfonyl) imide ($P_{222\,(2O1)}$ –TFSI) was our electrolyte of choice, and we based our choice on the information provided by a previous report that found quaternary phosphonium-type ionic liquids containing methoxy groups to have lower viscosity, higher conductivity, and higher thermal stability compared to their quaternary ammonium-type counterparts, [2] and to form fewer neutral ion aggregations compared to other ionic liquids, which means that a higher number of ions would be available to carry charge in such electrolyte.[3] The $P_{222\,(2O1)}$ –TFSI was purchased from Kanto Chemical Co., Inc. and used without further purification.

The thermal stability of the electrolyte was evaluated using thermogravimetric-differential thermal analysis (TGA-DTA) The procedure was performed by heating $P_{222\,(2O1)}$ -TFSI versus alumina as reference sample, at a rate of 5 °C / min under nitrogen flow using a Rigaku TG8120 analyzer.

**Electrochemical measurements**

The stability window of the electrolyte was determined from linear sweep voltammetry LSV. The experiment was performed using a Metrohm Autolab PGSTAT128N potentiostat in a three-electrode electrode cell consisting from platinum disk (outer diameter = 6 mm, inner diameter = 3 mm, electrode area= 0.942 $cm^2$) as the working electrode, platinum mesh as the counter electrode, and a silver wire as the reference electrode, with glass fiber filter paper (Advantech Co. GB-100R; thickness 0.38 mm) as separator. The measurement was performed at a sweep rate of 5 mV/s and temperatures up to 300 °C, in a constant-temperature chamber operated under an air atmosphere. The stability limits were determined using a conservative variation on the linear fitting method, as shown in in the example in Fig.S3.

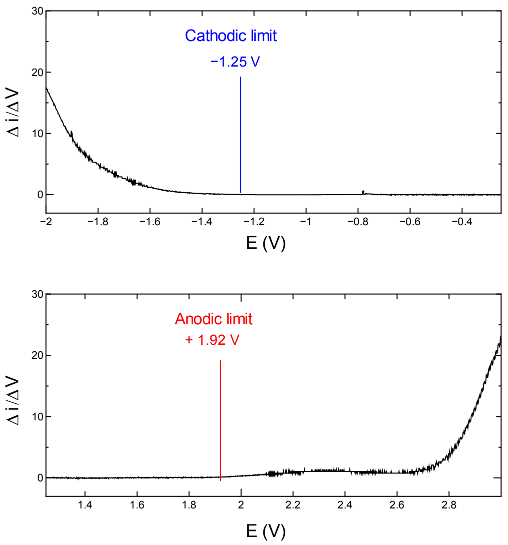


Fig. S3 Conservative linear fit approach to determine the electrochemical stability window of the electrolyte at 50 °C

Galvanostatic charging and discharging (GCD) and electrochemical impedance spectroscopy (EIS) measurements were performed in a Toyo System TOSCAT-3200 air-tight two-electrode cell, with the

SWCNTs or AC samples as both the working and counter electrodes, separated by glass fiber filter paper GB-100R. The free-standing SWCNT papers were used as electrode materials without the addition of a binder or conductive additives, while AC-based electrodes were fabricated by mixing AC with polytetrafluoroethylene (PTFE) binder with the respective mass ratio of 4:1, spreading the obtained paste on the current collectors, then oven-drying the electrodes at 60 °C. The cell was assembled in argon-filled glove box then taken outside to perform the measurement. GCD measurements were performed in the voltage range 0-1 at current densities 5, 10, 20, 40, and 80 mA/g. EIS measurements were performed at 0 V cell voltage, within the frequency range of 100 kHz – 1 mHz. Both GCD and EIS measurements were performed at temperatures between RT, 50, 100, and 150 °C. Fig. S4 and Fig. S5 show the GCD curves for the AC sample and the Nyquist plot measured for the AC sample at different temperatures.

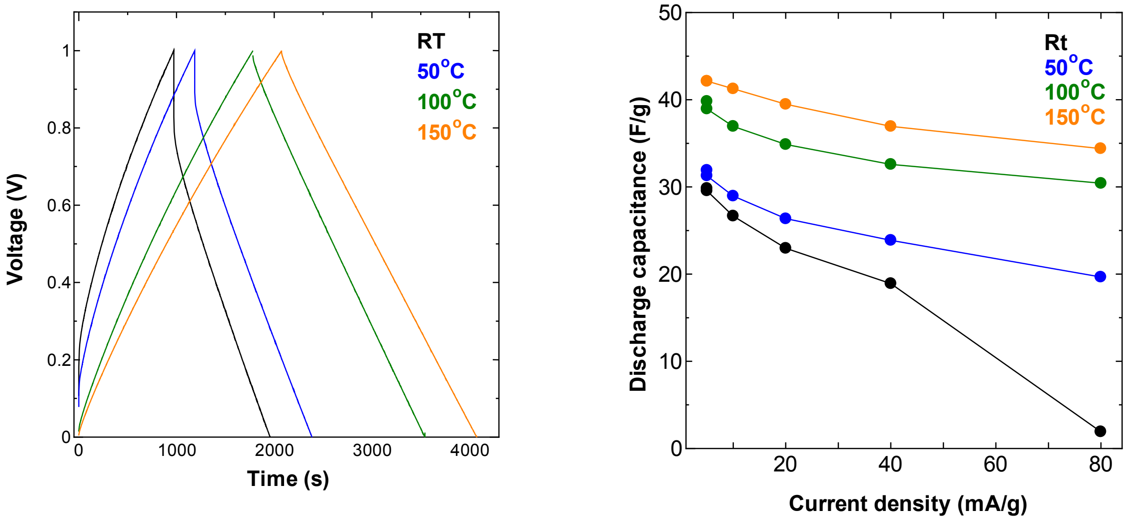


Fig. S4: (a) GCD curves and (b) Rate performance at different temperatures for the AC sample

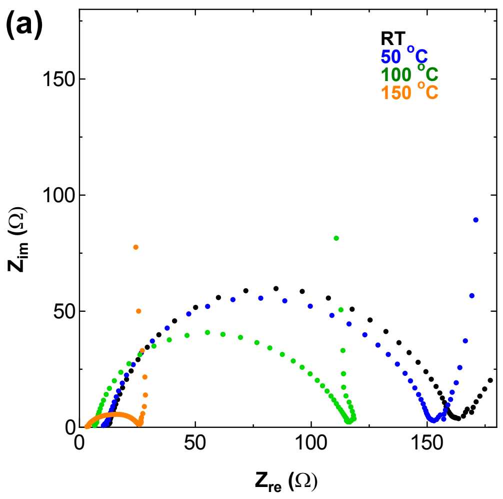


Fig. S5: Medium-high frequency range on the Nyquist plot for the AC sample

The EIS were fitted by Randles equivalent circuit model (Fig. S6) using EIS Spectrum Analyser software. The result is shown for SWCNT1.0 as example.

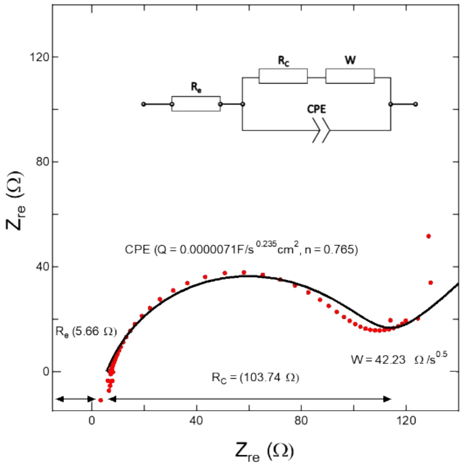


Fig. S6: Randles equivalent circuit model used for fitting the EIS data for SWCNT at RT

The ionic conductivity was calculated using the equation $\sigma = \kappa/Z$, where Z is the real part of the complex impedance (ohms) and $\kappa$ is the cell constant, considered to be unchanged over the temperature range,[4] and set to the value reported for $P_{222\ (2O1)}$ -TFSI at 25 C. The obtained ionic conductivity is shown in Fig.S7 below.

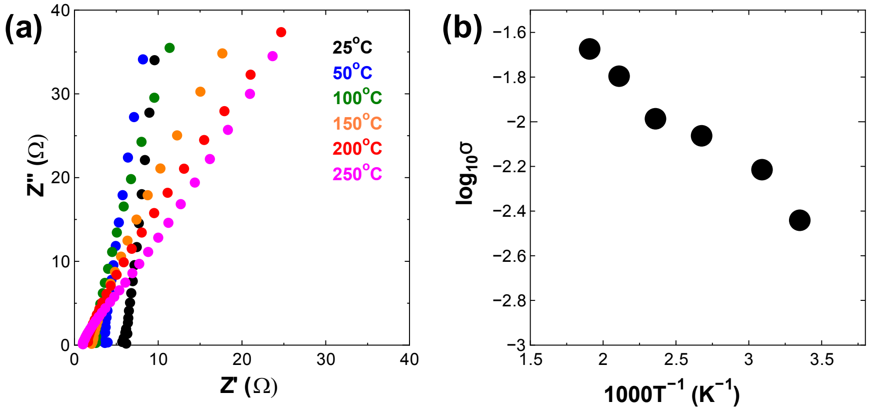


Fig.S7 (a) Nyquist plot and (b) ionic conductivity of $P_{222(2O1)}$-TFSI in the absence of the electrodes

## References


1 M. S. Dresselhaus, G. Dresselhaus, R. Saito and A. Jorio, Raman spectroscopy of carbon nanotubes, *Physics Reports*, 2005, **409**, 47–99.

2 K. Tsunashima and M. Sugiya, Physical and electrochemical properties of low-viscosity phosphonium ionic liquids as potential electrolytes, *Electrochemistry Communications*, 2007, **9**, 2353–2358.

3 V. L. Martins, N. Sanchez-Ramirez, M. C. C. Ribeiro and R. M. Torresi, Two phosphonium ionic liquids with high Li+ transport number, *Phys. Chem. Chem. Phys.*, 2015, **17**, 23041–23051.

4 L. Dagousset, G. T. M. Nguyen, F. Vidal, C. Galindo and P.-H. Aubert, Ionic liquids and γ-butyrolactone mixtures as electrolytes for supercapacitors operating over extended temperature ranges, *RSC Adv.*, 2015, **5**, 13095–13101.
